# Supplementary material for: In situ stable crack growth at the micron scale
Source: Nat Commun. 2017 Jul 24;8:108. doi: 10.1038/s41467-017-00139-w (PMC5524636; doi:10.1038/s41467-017-00139-w)
Supplement: Supplementary file 1 — Supplementary Information [file 41467_2017_139_MOESM1_ESM.pdf]

File Name: Supplementary Information
Description: Supplementary Figures, Supplementary Table, Supplementary Note and Supplementary References

File Name: Peer Review File
Description:

File Name: Supplementary Movie 1
Description: Video recorded by electron imaging during the test performed on the DCB labeled SC 3 of single crsytal SiC. After about 350 s, the tip is held still for another 300 s, during which the crack growth is not observed. Subsequently the tip is displaced of a small amount and then retracted. The DCB can be seen recovering its unloaded geometry at the end of the test. The tip displacement ramp, beam displacements and crack growth measured are presented in Fig.4 (a), (b) and (c). Frames of the video with a scale bar can be found in Fig. 3.

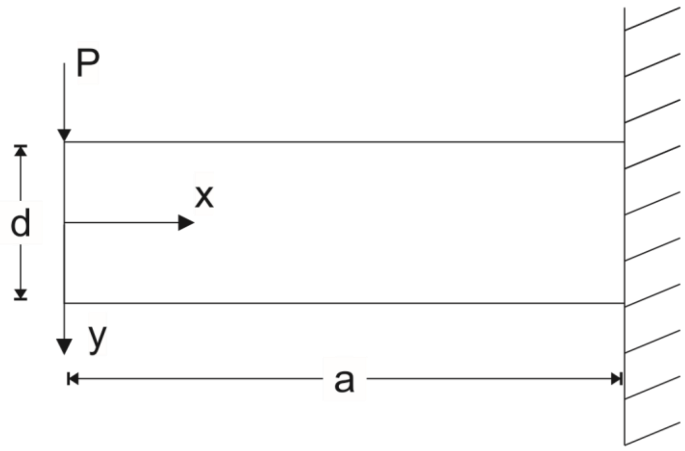

Supplementary Figure 1. Schematic of clamped cantilever loaded at the free end. Our double cantilever beam (DCB) system can be approximated as two individual clamped cantilevers of width d end-loaded by a load P, in which the clamp position is at the crack tip (i.e. at $x = a$) and the loading point is at the contact point between the wedge and the beam (i.e. at $x = 0$).

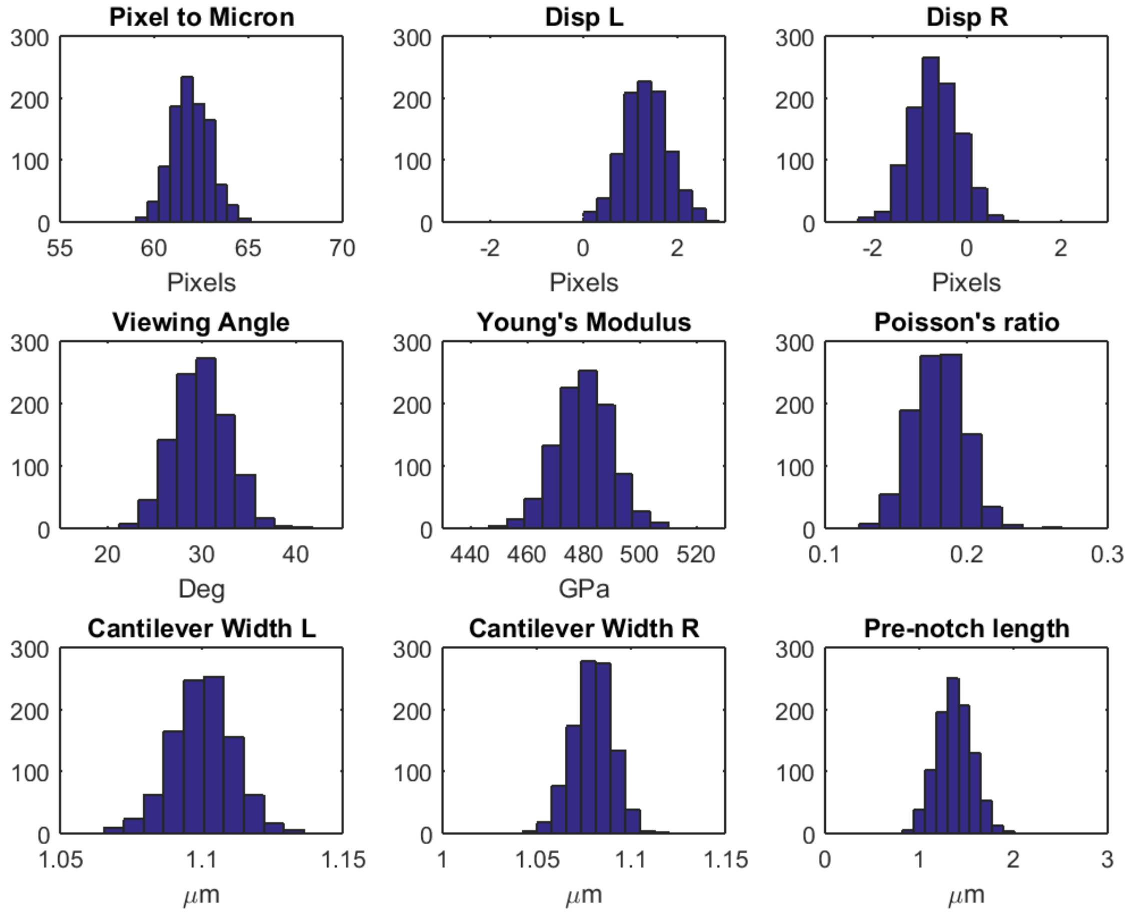

Supplementary Figure 2. Gaussian distribution of random inputs used for the Monte Carlo based error propagation analysis. The distributions shown are the ones used for one of the single crystal SiC DCB**.**

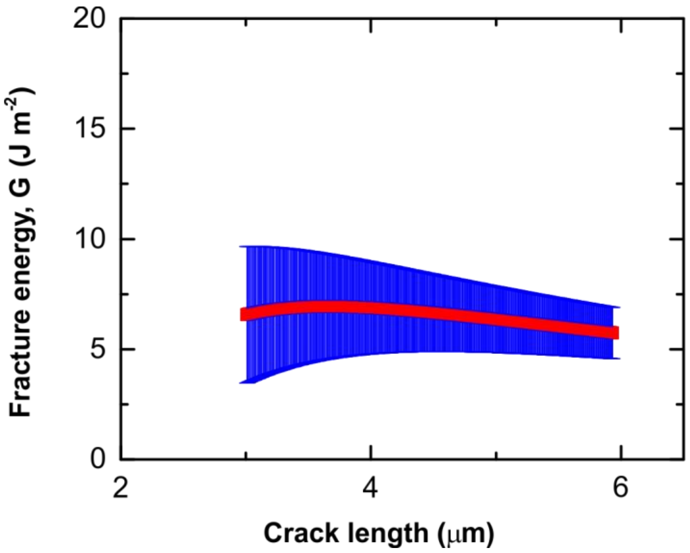

Supplementary Figure 3. Result of Monte Carlo based error propagation analysis on the measurement of fracture energy with crack length for one of the single crystal SiC DCB. The blue envelope represents the s.d., while the red line the mean value.

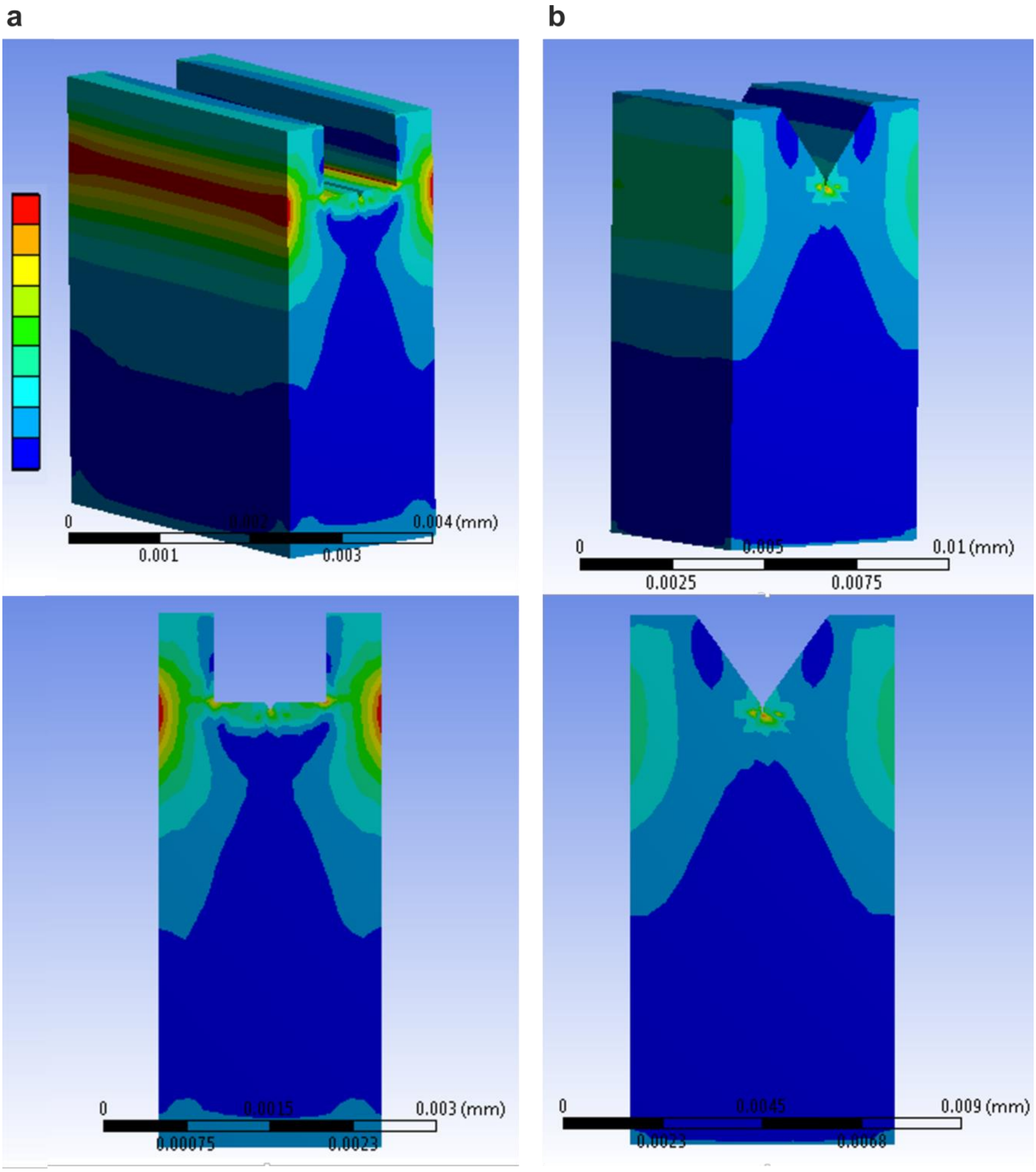

Supplementary Figure 4. Finite element analysis of different geometries and their effect on stress distribution. A static (i.e. elastic) structural finite element analysis (FEA) was carried out using the ANSYS software and setup considering a 10 mN load applied with a wedge tip. Red areas represent higher stresses, ~15 GPa in **(a)** and ~4 GPa in **(b)**, while blue lower stresses, ~0.5 GPa in **(a)** and ~0.2 GPa in **(b)**. Adjustment to the shape of central trough adjusts the location of the highest stress in the object away from the sides of the region. Despite being a crude evaluation of the actual stresses forming in the real DCBs tested, this analysis still provides useful information to understand the effect of modifications of DCB geometry. This information was used to improve the DCB fabrication and avoid undesirable failures of the arms. Scale bar: **a)** top 4 µm, bottom 3 µm; **b)** top 10 µm, bottom 9 µm.

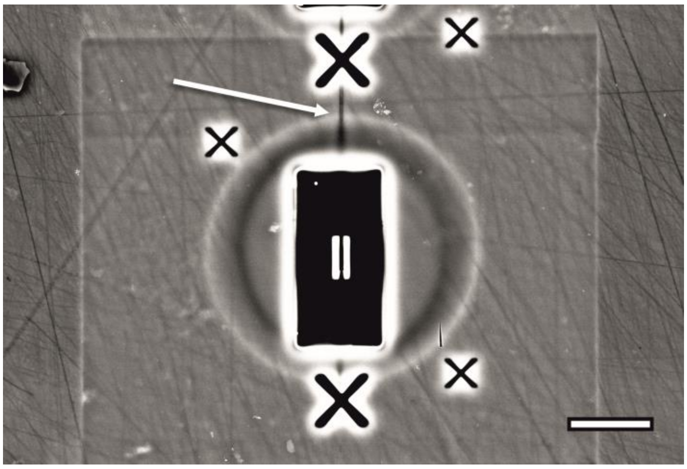

Supplementary Figure 5. High resolution backscatter top view image of the double cantilever beam milled along the glassy interface. The white arrow indicates the glassy interface confined between the two coupons of SiC. It is difficult to image the interface because its thickness is comparable to the resolution of the SEM. In addition, since the atomic masses between SiC and SiO_2 are similar they do not show a good contrast in backscatter mode. However, after the sample has been imaged with ions, due to preferential etching, the interface can be seen running through the centre of the sample as in the image. Scale bar: 10 μm.

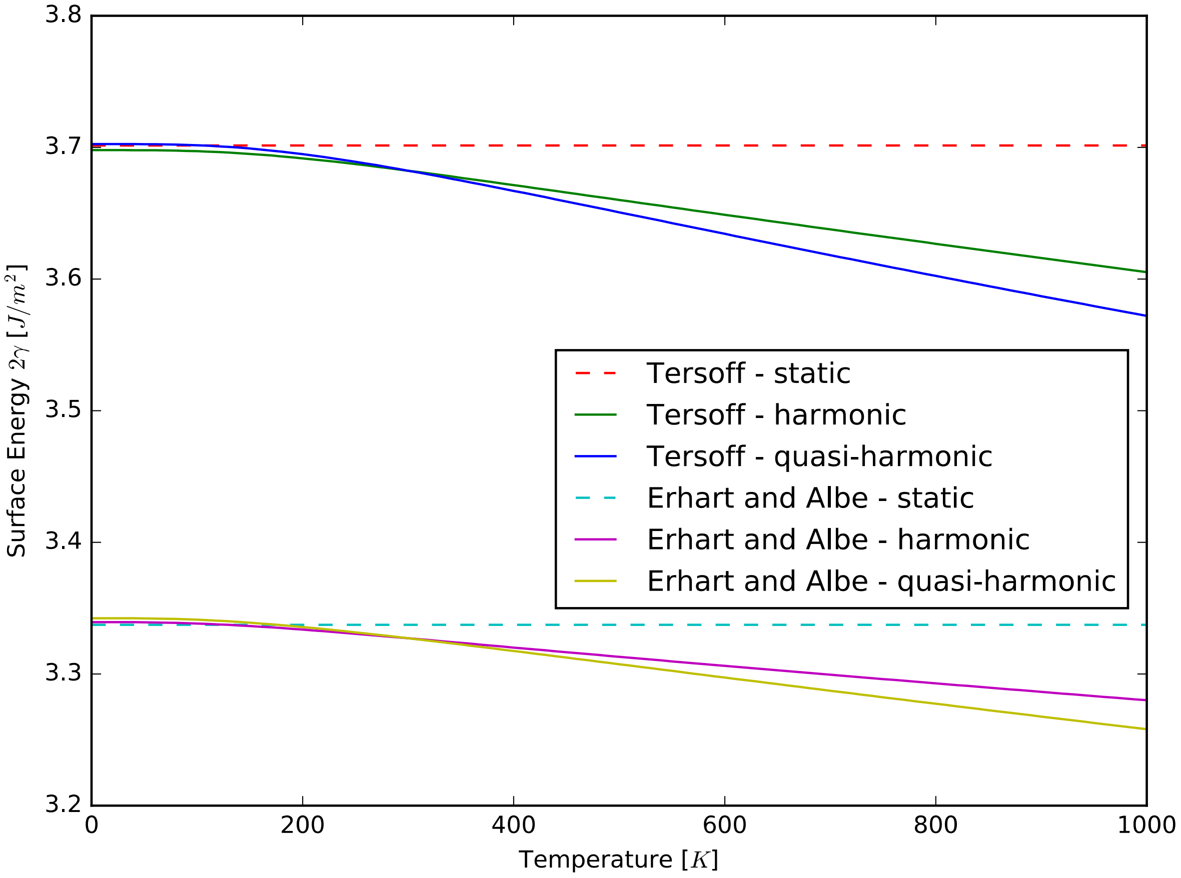

Supplementary Figure 6. Temperature Effects on Computed Surface Energies. Surface energies 2γ of the SiC 6H (0001) surface as a function of temperature using the harmonic and quasi-harmonic approaches. Screened versions of the Tersoff and Erhart & Albe interatomic potentials have been used[1]. For both models the reduction in surface energy at 1000 K is <4%.

Supplementary Table 1. Variables for the Monte Carlo based error propagation. Variables for the analysis with known errors were adjusted independently to have a Gaussian distribution with a known standard deviation (named SD in the table). The mean of each distribution was the measurement for each test and the standard deviations based upon experimental measurement error.

Quantity	Description	Uncertainty
Pixel to micron	pixel to micron conversion ratio used to measure distances from image analysis	1 pixel SD
Disp L	correction factor for left and right cantilever displacement to neglect initial recorded movements independent from pure bending and not corrected by frame registration	0.5 pixels SD
Disp R		0.5 pixels SD
Viewing angle	indentation axis tilt angle with respect to SEM stage used to correct for foreshortening	3° SD
Young's modulus	elastic modulus, E, used in equation (3)	10 GPa SD
Poisson's ratio	Poisson's ratio, ν, used in equation (3)	10%
Cantilever width L	left and right cantilever width, d, used in equation (3)	1%
Cantilever width R		1%
Pre-notch position	position of contact point between wedge and beam used to determine pre-notch length (and crack length by cumulative difference)	10 pixels SD

Supplementary Note 1

Timoshenko and Goodier[2] show that for a two-dimensional problem, when body forces are absent or are constant, the solution can be found in the integration of the differential equation:

$$\frac{\partial^4 \Phi}{\partial x^4} + 2\frac{\partial^4 \Phi}{\partial x^2 y^2} + \frac{\partial^4 \Phi}{\partial y^4} = 0 \tag{S.1}$$

With the stresses given by:

$$\sigma_x = \frac{\partial^2 \Phi}{\partial y^2};\ \sigma_y = \frac{\partial^2 \Phi}{\partial x^2};\ \tau_{xy} = -\frac{\partial^2 \Phi}{\partial xy}; \tag{S.2}$$

Solutions to the differential equation (S.1) can be found using polynomials of different degrees, such that by adjusting the coefficients it is possible to describe the solutions to the cantilever beam problem for different loading conditions.

Since a sum of polynomials is still a solution to equation (S.1) by the principle of linear superposition, it is the case that the bending of a cantilever loaded at its end can be solved by a combination of polynomials of second degree and fourth degree. We assume that the long upper and lower sides are free from forces, whereas the end load P is distributed parabolically as a shear force along the left face at $x = 0$.

A condition of pure shear is given by the following polynomial of second degree:

$$\Phi_2 = \frac{A_2}{2}x^2 + B_2 xy + \frac{C_2}{2}y^2 \tag{S.3}$$

with $A_2 = 0$ and $C_2 = 0$, for which by (S.2):

$$\sigma_x = C_2 = 0;\ \sigma_y = A_2 = 0;\ \tau_{xy} = -B_2; \tag{S.4}$$

In order to have a form that may cancel the uniform shear generated on the top and bottom surfaces by Φ_2, and that generates a parabolic shear on the ends that may equal P, the following polynomial

of fourth degree with all the coefficients except D_4 equal to zero can be used (which results in a normal stress σ_x that is proportional to y at a given location x along the length of the beam):

$$\Phi_4 = \frac{A_4}{4 \cdot 3} x^4 + \frac{B_4}{3 \cdot 2} x^3 y + \frac{C_4}{2} x^2 y^2 + \frac{D_4}{3 \cdot 2} x y^3 + \frac{E_4}{4 \cdot 3} y^4 \qquad \text{(S.5)}$$

Summing the two polynomials gives, from (S.2):

$$\sigma_x = D_4 xy;\ \sigma_y = 0;\ \tau_{xy} = -B_2 - \frac{D_4}{2} y^2; \qquad \text{(S.6)}$$

If we impose that the longitudinal sides at $y = \pm\frac{d}{2}$ are traction-free, we have:

$$\left(\tau_{xy}\right)_{y=\pm\frac{d}{2}} = -B_2 - \frac{D_4}{2}\left(\frac{d}{2}\right)^2 = 0 \ \rightarrow \ D_4 = -8\frac{B_2}{d^2} \qquad \text{(S.7)}$$

Therefore, noting that at the loaded end the resultant of the distributed shear stress must equal P, we can integrate $-\tau_{xy}$ to obtain B_2:

$$\int_{-\frac{d}{2}}^{\frac{d}{2}} -\tau_{xy} dy = \int_{-\frac{d}{2}}^{\frac{d}{2}} \left(B_2 - 4\frac{B_2}{d^2} y^2\right) dy = P \qquad \text{(S.8)}$$

$$B_2 = \frac{3}{2}\frac{P}{d} \qquad \text{(S.9)}$$

Thus, the stresses are:

$$\sigma_x = -12\frac{P}{d^3} xy;\ \sigma_y = 0;\ \tau_{xy} = -\frac{3P}{2d}\left(1 - 4\frac{y^2}{d^2}\right); \qquad \text{(S.10)}$$

Noting that the second moment of inertia for a rectangular cross section of unitary width is:

$$I = \frac{d^3}{12} \qquad \text{(S.11)}$$

we can write the stresses in the form:

$$\sigma_x = -\frac{Pxy}{I};\ \sigma_y = 0;\ \tau_{xy} = -\frac{P}{2I}\left(\frac{d^2}{4} - y^2\right); \qquad \text{(S.12)}$$

From the stresses we obtain the strains as:

$$\varepsilon_x = \frac{\sigma_x}{E} = -\frac{Pxy}{EI};\ \varepsilon_y = -\frac{\nu\sigma_x}{E} = \frac{\nu Pxy}{EI};\ \gamma_{xy} = \frac{\tau_{xy}}{\mu}$$

$$= -\frac{P}{2\mu I}\left(\frac{d^2}{4} - y^2\right); \tag{S.13}$$

where ν is Poisson's ratio, E is Young's modulus and μ is the shear modulus.

Hence, having both stresses and strains defined, it is possible to obtain the stored elastic strain energy by integrating the strain energy density over the volume V:

$$U_M = \frac{1}{2}\int_V \left(\sigma_x\varepsilon_x + 2\tau_{xy}\gamma_{xy}\right)dV = \frac{P^2a^3d^3}{24EI^2}\left(\frac{1}{3} + \frac{1}{5}(1+\nu)\left(\frac{d}{a}\right)^2\right) \tag{S.14}$$

The displacement δ of the beam at the neutral axis of the loaded end is given by[1]:

$$\delta = \frac{Pa^3}{3EI} \tag{S.15}$$

Therefore the load can be calculated knowing the displacement δ:

$$P = \frac{3\delta EI}{a^3} \tag{S.16}$$

Substituting (S.16) into (S.14):

$$U_M = \frac{3\delta^2 E}{8}\left(\frac{1}{3}\left(\frac{d}{a}\right)^3 + \frac{1}{5}(1+\nu)\left(\frac{d}{a}\right)^5\right) \tag{S.17}$$

Supplementary References

[1] Pastewka, L., Klemenz, A., Gumbsch, P. & Moseler, M. Screened empirical bond-order potentials for Si-C. *Phys. Rev. B* **87**, 205410 (2013).
[2] S. Timoshenko, J. N. Goodier. *Theory of elasticity*. (McGraw-Hill Book Company, 1951).
